# Salmagundi

**Published:** 1885-06

**Authors:** 


### SALMAGUNDI.

EDITED BY E. G. BETTY, D. D. S.

ABSTEMIOUS and facetious are said to be the only two words in which the vowels follow one another.

---

THE poem in the February number of the REGISTER, entitled "The Dental Chair," was written by J. W. Hurm, and originally appeared in the June number of the *Items of Interest* for 1880.

---

THE Fellows of the Royal College of Surgeons of Ireland, have voted to concur with the Council of the College in having the charter altered, so as to admit women to all diplomas of the college on terms equal with men.

---

A SOCIETY has been formed in Boston to look after the teeth of poor children. It might be as well, perhaps, to be sure that they have something in the way of food for them to use their teeth upon.—*Providence Journal.*

---

DR. MILLER, of Berlin has come to the conclusion from a series of experiments that, the comma bacillus found in the mouth is not identical with the cholera bacillus of Koch; this confirms the observation of others.

---

LABBE states that he has had favorable results with salicylate of soda in cases of sciatic and supra-orbital neuralgia, where quinine had failed. L. prescribes ʒij of salycilate of soda in the first two days; ʒj on the third day, before eating, with a little Vichy.

---

DR. WM. CLENDENIN is dead. This will be sad news to all of those who had the pleasure of listening to and profiting by his lectures on anatomy in the Ohio Dental College. A biographical sketch appears upon another page of the REGISTER, to which we invite the attention of all our readers.

---

IT is not at all unlikely that Dr. Watt may try his hand again at the amalgam question, cause why, the *Ohio State Journal* for March contains a curious article on "The Poisonous Effects of Amalgam Fillings," and the *Independent Practitioner* for May a reply to it in the nature of "Straining at a Gnat." Now's the time for Uncle Jerry to get in his work.

---

DR. WM. VAN ANTWERP, of Mt. Sterling, Ky., could not resist the attractions of the dog show held in Cincinnati during the first week of May, and spent a couple of days in the city enjoying himself thoroughly, for Van admires a good dog and a —, we were going to say 'gun,' but we won't, for fear folks might think he also took in the pigeon tournament.

---

THE *Sanitary Engineer* says there is one form of pneumonia that "does not depend on exposure to cold, but rather on exposure to a particular form of micro-organism which flourishes in damp and foul localities, and is the epidemic or contagious form of the disease which sometimes causes great mortality among the negroes in the South. The most important means of preventing this form of disease are those which we so often have recommended, viz. : cleanliness and fresh air."

---

Aconitine for Anæsthetizing Sensitive Dentine.—Dr. Anton Kozma, of Buda-Pesth, reports that a small piece of Japanese paper, three to four mm. square, saturated with three mgrm. of aconitine and pressed into the hollow tooth, previously cleaned out and dried, and then covered with an occlusive covering of gutta percha, anæsthetizes the sensitive dentine that renders operations on carious teeth so painful. The larger the quantity of aconitine employed the more quickly is the desired effect produced and the longer it lasts. The effect generally continues for from twenty-four to forty-eight hours.—*Medical Press.*

---

" A Concord (N. H.) doctor, has the laugh on a Boston professional brother. The latter declared the case of John W. Straw, hopeless, stating that cancer in his mouth was incurable. The sick man went home to New Hamshire, where a local physician looked into the case, stuck his forceps through a partially locked jaw, and extracted an ulcerated tooth. Straw is getting well."

There are two points in this paragraph not clear to us, because, possibly, they were not mentioned, viz. : It does not appear that the " local physician" was a dentist, and nothing is said about pyæmic complications.

---

How to Prepare Water for Drinking.—Dr. J. Wallace says : "My plan for years has been to have a tin can large enough to contain all the water usually drunk in a day, filled from the kitchen boiler when the cooking is in full swing and the water is actually boiling. This can is placed beside the filter until the next morning to cool, and then the filter is filled up, and the can is refilled with boiling water. The filtering completely effects the renovation of the water, and no one would suspect that it had been boiled. Many people would sooner drink pleasant-tasted, though vitiated water, than pure water which had lost its air, and to induce such people to boil water it is necessary to reærate it. Therefore boil first and then filter.—*London Lancet.*

---

HERE are two current news items which present a startling yet altogether logical comparison. Massachusetts last year enjoyed an almost total immunity from small-pox, but nine cases, with only one death, having been known to occur in the entire State in a twelve-month. In Kashmire the mortality during the year was of almost incredible proportions. The history of twenty-five families was recently taken, in which it appeared that, out of 190 persons, 100 died of small-pox. In Kashmire there is no vaccination; in Massachusetts it is absolute, compulsory, and as general and systematic as tax-paying. Peace hath its victories. If modern medicine had done no other single thing than demonstrate its control of one of the most terrible of human ills, it would be deservedly honored.

---

"DR. JESETT, Surgeon to the London Cancer Hospital, states that out of 2,227 cases of cancer known to him, the tongue was the seat of the disease in 190, or 8.5 per cent.; the breast in 697 cases, or 31.3 per cent. In 2,412 cases given by other surgeons, 8 per cent. were upon the tongue. Irritation is a cause of cancer, and one can not, therefore, be surprised that the tongue should be frequently affected. Carious and broken teeth, with rough, sharp edges, against which the tongue plays; the large quantity of tartar which collects upon the teeth; false teeth, especially when the plates fit badly, or are of inferior material; smoking, particularly the short clay pipe, with its jagged end; ardent spirits; hot condiments, chilies, pickles, etc., may all be placed among causes of tongue cancer.

Dr. Jesett's advice should be noted of all men. He says: "I would in all cases of suspicious ulcer of the tongue, if after ten day's or a fortnight's treatment it did not commence to heal or assume a healthy appearance, strongly advise the excision of the ulcer. Should it prove after its removal to be innocent, I should know my patient was not a bit the worse for its loss, while we would have been prevented from running the risk of further mischief."

---

The *Odontographic Journal* for April of the current year contains a full stenographic report of the toasts and speeches at the annual dinner of the Odontological Society of New York, February 18. Among the distinguished members of other professions present was Whitelaw Reid, Esq., editor of the *New York Tribune.* The following extract from his speech is pertinent and interesting, italics ours :

"I would like to say a word concerning the relations of the Press to the Profession of Medicine, in which dentistry is included. We have one; I am told it is an illegitimate relation, but it has nevertheless been, like other illegitimate relations, a very profitable and pleasant one. Most of you are thinking of Richmond, and those of you who are not thinking of Richmond I am sure are thinking of the honored name of Sheffield, a gentleman who has often paid us five hundred dollars for a single page advertisement; and this whole table together never did so much for the support of journalism. The only fly in this pot of ointment which has been set before us by the Odontological Society of New York to night is the fact that the *advertising* dentists are not here represented. *For the life of me I would never venture within their offices, but I am most delighted when they come to mine.* It does seem that there ought to be some compromise, some new digest of the ethics of your profession, by which it may come to pass that, when a man of an extra amount of brains gets up something which is of such extraordinary value, that it would be almost a crime to deprive the world of it, he may be permitted to pay us for telling the world of it. Let not your light be hidden under a bushel. If you had not put so many bushels on top of your heads, you would not be standing up to-night and making pleas for the dignity and honor of the profession of dentistry."

---

Early Dentistry.—At the date of my earliest recollection dentistry as now practiced was unknown. Teeth were extracted by regular practicing physicians generally, and their only outfit was an instrument known as the "turnkey" or "hawk's bill." It was constructed like a common nail gimlet with a movable hook at the end, which could be turned so as to seize upon any tooth whatever its position; then by a twisting motion the offending molar was rolled out. In country places where physicians were sparsely located, men in various occupations would keep a "turnkey" and perform the service. In one instance I knew of a lady who acquired the reputation of an expert at the business and had quite an extensive practice. The first artificial tooth which I ever saw was inserted by an itinerant dentist. It was secured upon a metal pivot or dowell and the pivot was forced into the stump of a decayed tooth. They were made from ivory or cattle's teeth and sometimes secured on wood instead of metal pivots. In 1835 there were in this city but three professional dentists. Not far from that time some one had secured two or more teeth to a metallic spring which clasped the adjoining teeth in such a manner as to hold them in position. That was thought to be a wonderful achievement and was proclaimed to the world. In that year there were slaughtered at one place near this city several hundred head of cattle for barreling, and cartloads of heads were piled near the slaughter-house. I saw one of those dentists approach them with a saw and sack and select such specimens as suited him. He then sawed off the under jaws containing the teeth, which he desired, and after filling his sack he put them into his buggy and departed. Somebody's mouth was doubtless ornamented with those teeth, and they took satisfaction in showing their "ivory." Since that period I shall not attempt to describe the inventions, progress, and improvements in the science, for I am utterly incapable. Instead of three, we now have twenty-three of the profession in this city.—*Hartford Post.*

---

THE TRADE IN SKELETONS.—Pedestrians on William street have had their attention called to the window of a dealer in surgical instruments, the past several months, by the complete skeleton of a human form sitting bolt upright in a chair at one corner.

Close beside it is a skull, on which is the placard, "Alas! Poor Yorick!"

When asked regarding the trade in this curious kind of merchandise, the dealer said:

"Sell many of them? Yes, sir; comparatively speaking we sell lots of them, although you don't see the article quoted in the market reports. Why, we usually are obliged to carry on hand a stock of from twenty-five to forty skeletons and a complete line of skulls. We sell them to museums, medical colleges, and physicians.

"The price of a skeleton varies from $30 to $60, according to its perfection and completeness. The demand is not so very large, from three to four hundred a year is probably the limit of the number of complete skeletons sold in this country. Our sales are about 150 a year.

"A curious feature of the trade is that all that are sold are imported. They come from Paris, where a regular business is done preparing them for the trade. Nearly all the skeletons imported to this country come to this city. Where do they get them? Oh, from morgues and the many cases of suicides that occur in France."

"Why can not they be procured in this country?" was asked.

"There is no establishment of the kind in this country that I know of, and the reason is that our people do not seem to have the facilities or inclination to go into this kind of business. Besides, it necessitates a costly outlay, and the business here is not of sufficient size to make it pay. A few years ago a Frenchman did come over here with the idea of starting the business. He did make a start in Philadelphia, but soon had to give it up after considerable loss, for he couldn't make it pay.

"That skeleton you noticed in the window is from France. It is a defective one, so we have had it on exhibition there. It attracts no little notice, and the other day a man came in here and, with sympathy expressed on his face, asked where that man used to live. We told him it was a Frenchman, whose first appearance this side of the Atlantic was probably in this manner. The visitor went away saying his sympathies were quite aroused over the poor skeleton sitting there in that painful position day after day, when he ought to be lying at rest in his grave."—*New York Mail and Express.*

